# Improving early identification of HIV-infected neonates with birth PCR testing in a large urban hospital in Johannesburg, South Africa: successes and challenges

**DOI:** 10.7448/IAS.20.01/21436

**Published:** 2017-04-10

**Authors:** Karl-Günter Technau, Louise Kuhn, Ashraf Coovadia, Sergio Carmona, Gayle Sherman

**Affiliations:** ^a^ Empilweni Services and Research Unit, Department of Paediatrics & Child Health, Rahima Moosa Mother and Child Hospital, Faculty of Health Sciences, University of the Witwatersrand, South Africa; ^b^ Gertrude H. Sergievsky Center, College of Physicians and Surgeons, and Department of Epidemiology, Mailman School of Public Health, Columbia University, New York, NY, USA; ^c^ Department of Molecular Medicine and Haematology, Faculty of Health Sciences, University of the Witwatersrand, National Health Laboratory Service, Johannesburg, South Africa; ^d^ Centre for HIV and STI, National Institute for Communicable Diseases, Johannesburg, South Africa

**Keywords:** HIV, diagnosis, neonate, polymerase chain reaction, birth, in-utero

## Abstract

**Introduction**: Timely diagnosis is necessary to avert early death in HIV-infected neonates. Birth PCR testing may improve early identification and facilitate access to care. We implemented a birth HIV diagnosis programme in Johannesburg, South Africa and present successes and challenges of the first two and a half years of operation.

**Methods**: Between June 2014 and December 2016, we sought to identify all HIV-exposed births and offer newborn HIV PCR testing before discharge after delivery. The programme identified newly delivered women who had tested positive during pregnancy and provided post-partum HIV antibody testing for women without recent negative results. HIV-positive women were required to consent for neonatal birth testing and asked to return a week later to obtain their results. Neonatal venous blood was sampled and tested at the national laboratory using Roche COBAS® TaqMan® HIV-1 Qualitative Test (Version 2.0). Non-negative results triggered active follow-up for confirmatory testing and appropriate treatment.

**Results**: Of 30,591 women with live births, 6864 (22.4%) were known to be HIV positive and an additional 221 women (1.4% of those tested) were identified during maternal postnatal testing. Of 7085 HIV-positive women, 6372 (89.9%) were interviewed and agreed to data collection, 6358 (99.8%) consented to birth testing for 6467 neonates and a blood sample was collected for 6377 (98.6%). If tested, 6210 (97.4%) tested negative, 91 (1.4%) positive, 57 (0.9%) revealed errors and 19 (0.3%) were indeterminate . Seven of the 19 neonates with indeterminate results and one with initial error result were found to be infected on subsequent testing yielding an intrauterine transmission rate of 1.6% (95% CI: 1.3–1.9). Sixteen (16%) of 99 infected infants were born to women (*n* = 221) identified during postnatal testing. With active outreach, 95/99 (96%) infected infants were initiated on antiretroviral therapy. Of 6261 neonates with negative results, 3251 (52%) returned to receive their test results.

**Conclusion**: Our programme successfully achieved high coverage and uptake of birth PCR testing and was able, with active tracking, to start almost all identified HIV-infected neonates on antiretroviral therapy. Implementation required additional staff for counselling, quality control and outreach. Return for negative results was low and neonates with indeterminate results required multiple repeat tests.

## Introduction

Timely diagnosis is necessary to avert early death in HIV-infected infants as these infants progress rapidly and almost half will die in the first two years of life without antiretroviral therapy (ART) [1]. Prevention of Mother-to-Child Transmission (PMTCT) programmes have been able to reduce the incidence of new HIV infections in infants dramatically [2,3]. Nevertheless transmissions continue to occur and, given the persistently high prevalence of HIV among child-bearing women, the numbers of new infant infections remain of major concern. There are several technical, biological and social reasons that hamper implementation of effective infant diagnosis programmes [4,5]. We, and others, have shown that HIV-infected infants are often identified late when presenting with advanced disease [6–8]. Failure to identify HIV-exposed infants earlier at scheduled follow-up testing has been found to be an important gap in the PMTCT cascade [9].

Diagnosis of HIV infection in infants and young children requires follow-up testing using HIV polymerase chain reaction (PCR) since infection can be acquired *in utero*, intrapartum and during breastfeeding. Until now, most programmes have selected six weeks as the age to offer the first PCR test [4]. This age was selected on pragmatic grounds to fit with the immunization schedule, at a point where almost all intrauterine and intrapartum infections should be detectable by PCR and aiming to minimize the number of PCR tests given the cost associated with the high volumes of tests required predominantly in low- and middle-income countries [4,10].

In settings, such as South Africa, with high rates of institutional deliveries (national rate 84%, City of Johannesburg >90%) [11] birth testing, now part of national guidelines, could achieve high coverage of the HIV-exposed births and avoid the loss to follow-up that has occurred with later testing [12]. Birth testing is expected to identify the majority of infected children as, with more effective PMTCT, intrapartum infections are less common [13–15]. Importantly, birth testing allows for identification of intrauterine-infected neonates who may die before later testing [1,6,14,16].

We implemented a universal birth HIV diagnosis programme at Rahima Moosa Mother and Child Hospital (RMMCH) in Johannesburg, South Africa, in June 2014. Here, we present the successes and challenges of the first two and a half years of the programme to highlight the logistical challenges of implementation.

## Methods

We implemented a pilot universal birth HIV PCR testing programme at RMMCH in order to diagnose HIV-infected neonates as early as possible and initiate ART. At this time, the South African public health services supported PCR testing at the six weeks immunization visit at primary health care clinics. In 2013, guidelines expanded to recommend that PCR testing also be offered at maternity services for high-risk HIV-exposed neonates, including those who were preterm or low birthweight and in June 2015, this was amended to universal birth testing of all HIV-exposed neonates [12]. At this time, PMTCT guidelines in South Africa supported Option B + i.e. initiation of ART for all HIV-positive pregnant women upon diagnosis and advised six weeks of daily nevirapine prophylaxis for all HIV-exposed infants.

We aimed to offer HIV PCR testing for all HIV-exposed neonates born at RMMCH or presenting hours after birth if birth had occurred at home or on the way to the hospital (born before arrival). Our site had already established a maternal HIV testing programme at delivery for non-positive women in the antenatal, delivery and postnatal wards [17]. We define “postnatal” as the period in hospital after birth (well and with mother in obstetric ward or under observation/admitted to nursery). Post-Caesarean section mothers stayed for three days while post-vaginal delivery stays ranged from 6 to 24 h. Well neonates born overnight were seen the following day in the postnatal wards while ill neonates were attended to by the treating clinicians. Prior to January 2015, women of unknown HIV status or with negative HIV test results more than 6 weeks earlier were offered testing [12]. From January 2015, all non-positive women presenting for delivery were offered testing. Two counsellors screened all women in the postnatal wards to identify known HIV-positive women and to offer maternal HIV testing for those who qualified. Maternal testing was done using ADVANCED QUALITY^TM^ rapid anti-HIV 1&2 test (InTec Products Inc., Xiamen, China) for screening, and ABON^TM^ HIV 1/2/O Test (Abon Biopharm, Hangzhou, China) for confirmation.

Two additional counsellors interviewed all HIV-positive women regarding their medical and obstetric history and HIV treatment before discussing informed consent for neonatal PCR testing. These data were recorded on paper forms that were captured electronically into a REDCap database maintained to provide routine data on HIV-related services at RMMCH [18]. Separate signed informed consent for both routine data collection and for neonatal testing was obtained. Blood was drawn from neonates within hours after delivery by venipuncture. Two paediatric phlebotomy-trained nurses were responsible for Ballard scores to assess gestational age and venesection [19]. Partial weekend and public holiday cover by the study staff occurred from August 2014 to June 2016.

Neonatal venous whole blood was collected in 0.5 ml ethylenediaminetetra-acetic acid (EDTA) tubes and sent to the National Health Laboratory Service’s central HIV PCR laboratory for testing on Roche COBAS® TaqMan® HIV-1 Qualitative Test Version 2 (Roche Molecular Systems, Inc., Branchburg, NJ). Whole blood was stored as dried blood spots (DBS) at −80°C as a precaution if additional testing was required. All samples were recorded on a sample tracking log sheet by the phlebotomist using a unique tracking barcode that matched each individual patient record. The laboratory alerted the clinical team electronically to all non-negative HIV PCR results, including errors, as soon as these results became available. Indeterminate HIV PCR results were defined by standardized laboratory test criteria and represent either low-level viremia or false positives.

Mothers were invited to the hospital’s out-patient HIV clinic on a Monday one week post-delivery to receive their neonate’s test result. Two data capturers prepared all patient files in advance of the return date by printing results. Mothers of neonates with negative birth PCR results were advised to go for follow-up testing at their local primary health care clinic as per national guidelines. Active outreach efforts were made to ensure that all mothers of neonates with non-negative results received the results.

For infants with positive and indeterminate initial PCR results, repeat qualitative PCR and viral load testing using COBAS® AmpliPrep/COBAS® TaqMan® HIV-1 test (Roche Molecular Systems, Inc., Branchburg, NJ) was done on a second blood sample to confirm the neonate’s HIV status. ART was offered through the hospital’s paediatric HIV service for all presumed HIV infected neonates. ART was initiated as soon as possible after one positive result prior to confirmation. Where a neonate’s initial result was unavailable due to a sample or laboratory error, they were re-bled where possible or their stored DBS sample was tested.

De-identified data from June 2014 through December 2016 were extracted for analysis. Data entry was verified and cross checked and data cleaning queries were run regularly. Descriptive statistics of the coverage and outcomes of the programme were analyzed using SAS (Version 9.4, SAS Institute Inc., Cary, NC).

The National Department of Health provided permission for universal HIV PCR testing of all HIV-exposed neonates at RMMCH in May 2014 prior to the inclusion of this recommendation in the national guidelines. The Human Research Ethics Committee of the University of the Witwatersrand approved the use of data for observational evaluation of birth testing (M140555 and M140639).

## Results

**Figure 1.**
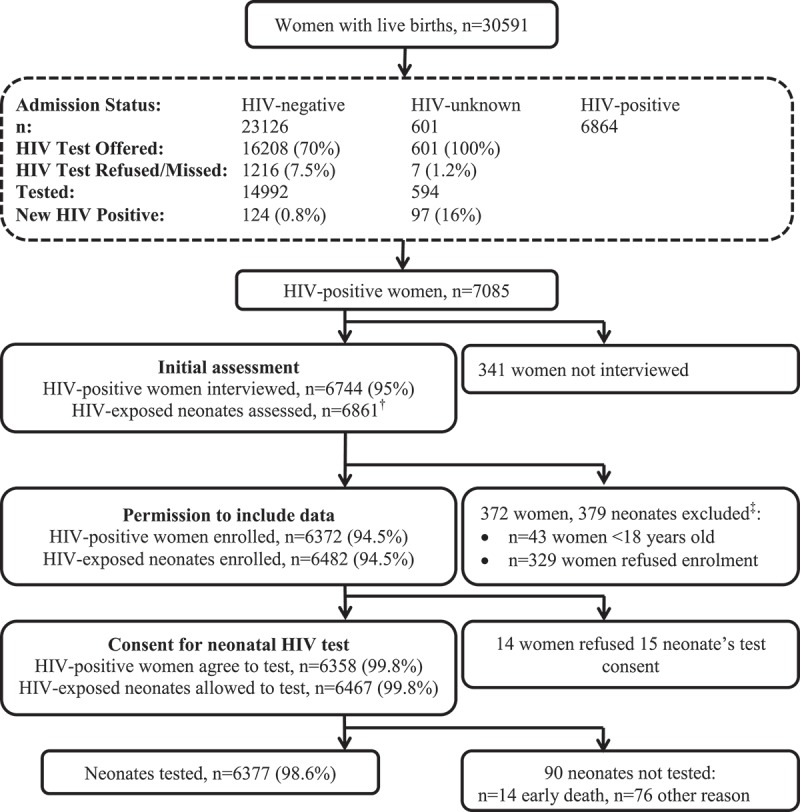
Identification of HIV-positive mothers and subsequent coverage and uptake of neonatal testing. †includes 111 second twins and three second and three third triplets. ‡ 318 (83.9%) of the 379 excluded neonates had a birth HIV PCR test performed with three testing positive, 312 negative, one indeterminate and two error results.

Between June 2014 and December 2016, 30,591 women and their neonates were admitted to the postnatal wards after having delivered at the hospital or being born before arrival. Of the total, 6864 (22.4%) women were known to be HIV positive. HIV antibody tests were offered to 16,809 (68.3%) of the 23,727 non-positive women and 15,586 (92.7%) of these agreed to test. An additional 221 (1.4%) of 15,586 women tested were found to be HIV positive as part of postnatal testing. Thus, the programme identified 7085 HIV-positive women, 3.1% of whom were newly identified during postnatal maternal testing (Figure 1). The average age of the HIV-positive women was 30.4 years (interquartile range [IQR] 26–34) and 22% had <12 weeks of ART prior to delivery.

**Figure 2.**
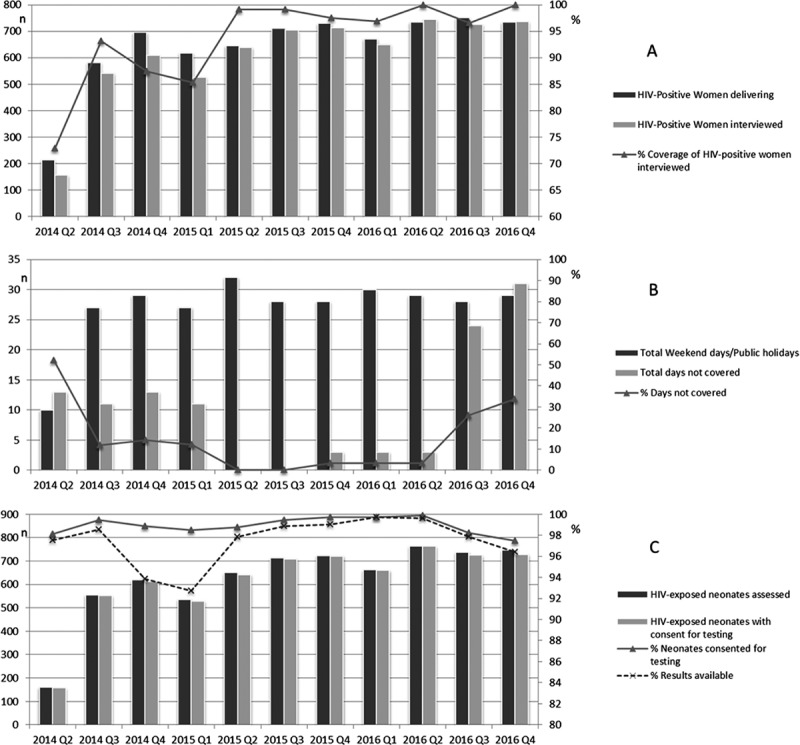
Enrolment trends over time – Panel A: Coverage of HIV-positive mothers interviewed, Panel B: Study staff capacity to cover birth testing daily, Panel C: Coverage of birth testing. Q = quarter; Q1 = January-March; Q2 = April-June; Q3 = July-September; Q4 = October-December.

Counsellors interviewed 6744 (95.2%) of the 7085 HIV-positive women and 6372 (94.5%) met eligibility and agreed to routine data collection. Of these, 6358 (99.8%) agreed to birth PCR testing for their 6467 neonates (109 neonates part of multiple birth) of whom 6377 (98.6%) actually had blood drawn for the test. Of the 90 neonates not tested, early death (median age 1 day [IQR: 0–2]) due to prematurity precluded testing in 14 neonates (median birthweight 745 g [IQR: 665–830]) and the remainder were due to logistical limitations including lack of phlebotomy capacity or inability to wait for phlebotomy (Figure 1). Taking all these steps in the cascade into account, we estimate that 6695 (97.6%) of the 6861 HIV-exposed neonates had a blood sample collected for PCR testing. Coverage of the birth testing programme (defined as the proportion of HIV-positive mothers interviewed each quarter) varied over time (Figure 2A). Coverage improved rapidly at the onset of the programme from 72.9% reaching 99.1% in the second quarter of 2015. However, coverage at the end of 2014 declined before improving in the second quarter of 2015. This is most likely explained by reduced study staff capacity to cover birth testing. The decline did not occur towards the end of 2015 or 2016 because staff capacity was boosted by routine ward staff taking responsibility for the programme. Figure 2B displays the number of weekend or public holiday days in each quarter as well as the total number of days in each quarter not covered by study staff. Uptake (defined as the proportion of HIV-exposed neonates whose mothers consented to HIV testing if interviewed) remained high and fairly consistent across all months. However, the proportion of consented neonates who had blood taken declined at the end of 2014 and again towards the end of the study period (Figure 2C).

**Figure 3.**
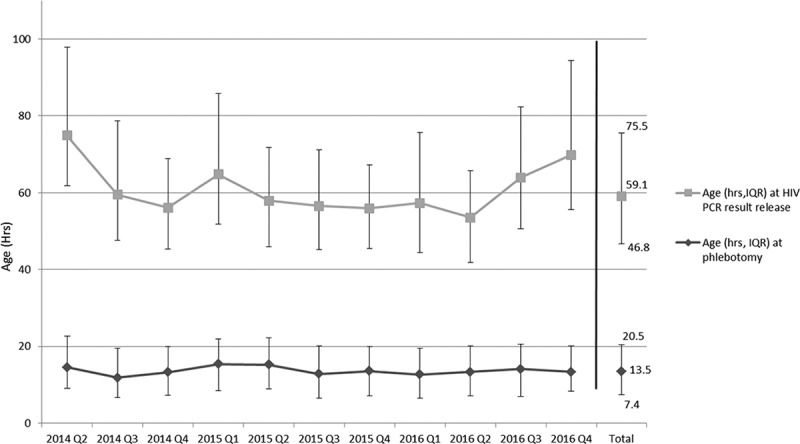
Age (hours) of neonate at phlebotomy for first HIV PCR test and at HIV PCR result release. hrs = hours, IQR: interquartile range. Q = quarter.

Blood was drawn from the 6377 HIV-exposed neonates at a median age of 14 h (IQR 8–21) and results were released to the site at a median age of 59 h (IQR 47–76 h; Figure 3). In total, 95% of neonates were tested before 30 h and 99% before 61 h of life. The age at the time of sample collection did not differ significantly between HIV-infected and -uninfected neonates. Most neonates (97.6%) had blood drawn for PCR after the first dose of nevirapine prophylaxis was administered at median age of 25 min (IQR: 10–114). The median time between phlebotomy and result release from the laboratory to the clinical site was 44 h (IQR: 32–58 h), with non-negative results released a median of five hours later than negative results (*p* = 0.0005).

**Figure 4.**
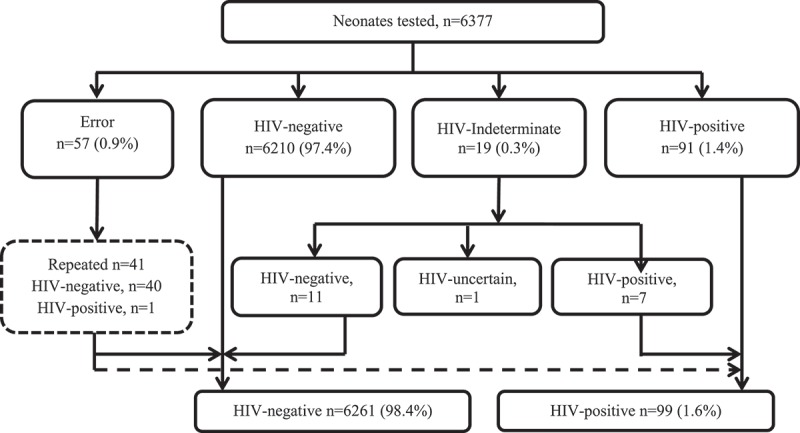
Initial birth HIV PCR test results and subsequent HIV infection status after repeat testing of neonates.

Of the 6377 neonates tested, 6210 (97.4%) samples tested negative, 91 (1.4%) positive, 57 (0.9%) yielded errors and 19 (0.3%) were indeterminate. For the 57 neonates with birth tests that failed to yield results, 41 (72%) had repeat tests performed on the stored DBS sample in 24 (59%) and on repeat sampling during a later visit in 17 neonates. Forty tested PCR negative, one was positive. Indeterminate results comprised 19 of 110 (17%) non-negative PCR results and required repeat testing to establish a definitive diagnosis. In total, 7 of the 19 neonates who initially tested indeterminate were subsequently classified as HIV infected. Five of them had viral load >1000 cps/ml (copies per millilitre) and qualitative PCR-positive results on repeat testing, two had repeat qualitative PCR-positive results. One neonate died due to complications of prematurity before repeat testing could be performed. The remaining 11 neonates with initial indeterminate results tested HIV PCR negative on subsequent tests, had no detectable viral load on any sample and were subsequently classified as HIV uninfected (Figure 4). In sum, we estimate the intrauterine transmission rate in this cohort to be 1.6% (99/6360–95% CI: 1.3–1.9). Sixteen (16%) of these 99 infected neonates identified at birth were born to 221 women newly diagnosed as part of the postnatal maternal testing programme.

Of the 99 HIV-infected neonates, 88 (89%) returned to the site and underwent confirmatory testing and eight (8%) received follow-up testing at other clinical sites. Three neonates were lost to follow up with no records of confirmatory HIV testing or contact with the health service. Of the 96 infected neonates who underwent confirmatory testing at any site, all but three were confirmed either with a subsequent positive PCR or with a detectable viral load test. These three neonates tested indeterminate or negative on confirmatory PCR tests and are being monitored closely. The median viral load among 94 neonates with results was 4.4 log cps/ml (IQR: 3.3–5.3 log cps/ml, range: 1.8–6.7 log cps/ml).

Of the 99 HIV-infected neonates, 88 (89%) returned to site to receive their test results at a median of 8 days (IQR 6–13 days, range 2–103 days) at which time ART was initiated and blood was drawn for confirmatory testing. Of the eleven neonates who did not follow-up on site, seven are known to have initiated ART at other clinical sites between 40–99 days. Thus, 95 of 99 (96%) HIV-infected neonates identified as part of the birth testing programme initiated ART. Of the 6261 neonates with negative birth HIV PCR results, 3251 (52%) received their test results on site at a median age of 10 days (IQR: 8–12, range 1–381).

## Discussion

In the first two and a half years of operation, our universal birth PCR testing programme was able to achieve >90% coverage of the HIV-exposed neonates delivered at Rahima Moosa Mother and Child Hospital. Almost all (99%) HIV-positive women consented for neonatal testing and drop-offs in the cascade were primarily due to our capacity to reach the identified HIV-positive women prior to discharge. Our capacity to reach the identified HIV-positive women varied considerably by quarter and was determined by our ability to ensure that study staff were present on all days in the month, including weekends and public holidays. In quarters where we had study staff coverage on weekends and public holidays, coverage exceeded 98% and it is encouraging that coverage was maintained at a high level despite the study staff withdrawing on the weekends as a result of routine staff conducting birth testing. In June 2015, universal birth testing of all HIV-exposed neonates was adopted by the South African PMTCT guidelines [12,20]. While the majority of women deliver in a healthcare facility in South Africa, birth PCR testing programmes will need to ensure that they can provide adequate staff coverage on all days, including weekends and public holidays, to achieve the full potential of birth testing for early infant diagnosis.

Our site had already established a maternal HIV testing programme at the time of delivery as advised by South African policy [12,20]. We were able to leverage this programme to identify the HIV-exposed neonates who needed testing. Maternal testing coverage for previously negative women was only 70% because during the initial study period guidelines did not require women with recent negative results to undergo testing. Although a relatively small proportion (3.1%) of HIV-exposed neonates was identified from newly diagnosed mothers, this group contributed 16% of all HIV-infected neonates. This highlights the importance of maternal testing on newly delivered women with non-positive results to identify high-risk infants, either HIV infected and in need of ART or HIV exposed and in need of PMTCT. Many of these newly identified HIV-positive women may represent incident infections at high risk of transmitting and, by definition, none will have accessed PMTCT [17,21–23].

We were pleased to observe a low intrauterine transmission rate of 1.6%. This is reassuring confirmation of the excellent PMTCT coverage in this population with the effective Option B+ strategy of initiation of maternal ART upon diagnosis. Given the logistics in the postnatal wards of RMMCH, it was not possible to collect a blood sample from neonates prior to the first nevirapine prophylaxis dose. Nevertheless we used the current state-of-the-art qualitative HIV PCR test from Roche (COBAS® TaqMan® HIV-1 Qualitative Test Version 2) which has been optimized with improved sensitivity compared to prior versions [24]. A limitation of our programme was the inability to provide systematic follow up of neonates who tested negative at birth to determine the rates of infections detected later as well as the coverage rates of later tests.

Indeterminate HIV PCR results accounted for almost a quarter of non-negative birth results and required follow-up with multiple subsequent tests to determine the infant’s infection status. Indeterminate PCR results may signify true infections with low levels of HIV available or may be false positives [13]. The additional tests and consultations to resolve these diagnostic dilemmas added costs and complexity to the programme.

We were able to ensure that almost all (95/99) neonates who were found to be HIV infected initiated ART, most in the first two weeks of life. However, this entailed active outreach efforts as well as liaison with other clinical sites. Without active tracking, only 52% of the mothers of infants with birth PCR-negative results returned to receive the results. This was despite clear counselling offered as part of the programme. Further efforts are required to facilitate return of results. “Results for Action” reports listing HIV PCR results for a facility are emailed weekly and being used to trace infants with non-negative results. Point-of-care testing offers an opportunity for immediate result return before discharge. To reduce the pressure on staff at the time of delivery and reduce the counselling required, antenatal visits should be used to prepare pregnant women for neonatal birth and follow-up testing. Additional counselling at delivery will be required to re-inforce the importance of early infant diagnosis.

In our programme, turnaround time of the PCR results was a median of 2 days and at a median neonatal age of 59 h. The 5-h delay in turnaround time for non-negative results is likely due to quality assurance procedures for positive results and repeat test runs for indeterminate results and unlikely to impact on clinical management. At our site, like in most maternity services in South Africa, the majority of neonates will have been discharged from the healthcare facility by at least 48 h of age. Hence, neonates with non-negative results need to be urgently located for confirmatory testing and ART initiation. Point-of-care testing at delivery may mitigate losses that occur due to failure to return for results. In the meantime, active outreach efforts are essential to ensure that infants with non-negative results are located and engaged in care.

For the infants we were able to manage at our site, we elected to initiate ART presumptively based on a single positive PCR result. We aimed to draw a second blood sample on the day of ART initiation for confirmation. The motivation for this is the growing appreciation of the importance of early initiation of ART in infants [25]. All of the infants we managed were confirmed on at least one qualitative PCR or with detectable HIV RNA on at least one viral load test.

Since HIV PCR testing on a blood sample collected <48 h of life is thought not to be able to detect an intrapartum infection, follow-up testing is necessary to detect these and postnatal breastfeeding-acquired infections. Given the cost of PCR tests particularly at high volumes, decisions need to be made about the frequency and timing of these subsequent tests whilst balancing the need for this vulnerable population of exposed and infected neonates to receive appropriate interventions[10].With the addition of birth testing, the South African national programme now advises that the second PCR test be scheduled at 10 weeks of age, 4 weeks later than previously recommended for the first PCR. With PMTCT, the ratio of intrauterine:intrapartum infection shifts [14], with an increasingly small proportion of infections occurring intrapartum. In 2014, the 6-week HIV transmission rate for Gauteng, the province where RMMCH is situated, was 1.6% (National Health Laboratory Service). Since our presumed intrauterine transmission rate was 1.6%, we estimate that we detected the majority of the combined toll of intrauterine and intrapartum infections. Therefore, a birth testing programme combined with 10-week repeat testing should not undermine the gains of the earlier 6-week testing programme despite the delay in detecting intrapartum infections. High birth testing coverage may prevent losses to follow-up and mortality of infected neonates. Better understanding of the extent to which PMTCT interventions (e.g. extensive maternal ART and daily dose nevirapine) may affect the performance of different infant diagnostic tests would be helpful to select optimal time points for later testing.

An important limitation is that our birth testing programme was implemented in the context of a study where additional resources were provided for counselling, follow-up and quality control. Therefore, our outcomes may not be generalizable to routine rollout at other hospitals in South Africa and elsewhere. We worked closely with the routine ward staff in the maternity service but provided additional counsellors and phlebotomists as well as a study team who provided management and quality control. The challenge to maintain coverage of the birth testing programme over weekends and public holidays reduced over the study period as the programme was incorporated into routine care. Efforts to incorporate the tracking and data capturing systems into routine care are ongoing whilst shifting study staff away from full service delivery and into training and supervisory roles. Innovative ways of incorporating other existing staff into the programme are being explored. We required written informed consent for neonatal birth testing which arguably might not be required in routine service. Consent for neonatal testing was extremely high (99%) but even were formal verification of consent not required, the process of explaining birth testing and the necessity for further follow-up and engagement in care is essential in a routine programme as well. The other staff-intensive components of the study, including quality control (e.g. ensuring bloods and results matched to the correct infant) as well as tracking the infants with non-negative results, engaging and retaining them in care, are also essential components of any routine programme.

## Conclusions

We have demonstrated that universal birth PCR testing of HIV-exposed neonates can be successfully implemented in a large urban hospital and can lead to early identification of HIV-infected infants and early initiation of ART. In our experience, it is the human resources that are critical to implementing the programme and achieving retention in the PMTCT cascade. Important staffing considerations include a strong counsellor presence covering seven days a week including public holidays, to provide three tiers of counselling: (1) maternal screening and testing for all delivering women, (2) maternal interviewing and counselling for all known and newly diagnosed HIV-positive women including taking consent for neonatal HIV testing and (3) follow-up counselling to provide results and encourage follow-up tests. Staff to perform neonatal phlebotomy, or collect dried blood spots, liaise with the laboratory, assemble results, and maintain adequate records are also required. Systems to encourage further postnatal testing of infants with negative results as well as to ensure timely follow-up and linkage to care of all neonates with non-negative results are crucial.
